# Supplementary material for: Improved cookstoves to reduce household air pollution exposure in sub-Saharan Africa: A scoping review of intervention studies
Source: PLoS One. 2023 Apr 27;18(4):e0284908. doi: 10.1371/journal.pone.0284908 (PMC10138283; doi:10.1371/journal.pone.0284908)
Supplement: S2 Table — (DOCX) [file pone.0284908.s002.docx]

# Improved cookstoves to reduce exposure to household air pollution in sub-Saharan Africa: A scoping review of intervention studies.

Eunice Phillip, Jessica Langevin, Megan Davis, Nitya Kumar, Aisling Walsh, Vincent Jumbe, Mike Clifford, Ronan Conroy, Debbi Stanistreet

**S2 Table. Available descriptions of ICS examined in this review**

| **ICS name & reference** | **Description** |
| --- | --- |
| Chitetezo stove | The Chitetezo stove is a simple clay cookstove for burning solid fuels that aim to reduce exposure to household air pollution by burning fuel more efficiently than an open fire, reducing fuel consumption and producing fewer waste combustion products. |
| Energy-efficient cookstoves | Unnamed cookstoves provided by Farmers Helping Farmers. It is equipped with a chimney to carry much of the smoke outside the house, and that reduces the amount of wood required for heating and cooking. |
| EcoChula | Electric fan-assisted gasifier with ceramic chamber. |
| EcoZoom Dura cookstove | The ‘rocket’ concept uses an internal ‘chimney’ in the stove that directs air through the burning fuel (usually biomass) and encourages the mixing of gases and flame above it. Precise internal stove dimensions are used to achieve high combustion efficiency and transfer heat to the cooking pot. |
| Envirofit | Improved rocket with metal alloy chamber. |
| Firewood Jambar | Portable cookstove with a fired clay combustion centre enclosed by a metal casing. Owing to basic design improvements of the Jambar compared to traditional stoves, the wood fuel burns more efficiently, and the heat is better conserved and focused on the cooking pot. |
| Gastov: | The gasifier cooking stoves are gas burners that produce their gases from dry solid biomass. Gasifiers transform firewood into energy in four stages, namely drying, pyrolysis (carbonisation), gasification and gas combustion. Encouraged to light the stove outside. |
| Gyapa wood stove | Unvented wood-burning rocket cookstove. |
| Hifadhi | A locally produced improved cookstove with two compartments comprising an air entrance at the bottom with space for fuel and a combustion chamber on top. The inner walls are made of cement, which is enclosed by an outside layer of galvanized steel—distributed to farmers by the Climate Pal-Kenya project. |
| Kuniokoa- BURN | A natural draft wood-burning stove. The stove achieves IWA performance tiers of 3 for emissions, 2 for fuel efficiency, 2 for indoor emissions and 3 for safety, where the tiers range from 0–4. |
| Mirt | A local household and institutions made stove. Constructed with mortar (Sand/cement mixture). Consists of six parts which are assembled by the end-users. Consists of a combustion chamber, chimney, inlet for biomass fuel and air. It can be used to cook or boil water while simultaneously making local Injera dishes. |
| Modern charcoal Jiko | It is constructed of metal, with a stainless-steel combustion chamber and an ashtray that can be adjusted to regulate primary airflow. The traditional Jiko has a metal-clad ceramic liner, three-pot supports, and metal legs. |
| Philips model HD 4012 | Forced-draft advanced combustion cookstove with a solar panel to charge the battery for the stove fan |
| Philips: South Africa, Johannesburg |  |
| Prakti | Double pot rocket with chimney and steel alloy chamber. |
| Rocket mud stove | Based on the “rocket gas burner which combines both clean-burning and optimized heat transfer characteristics. Through more efficient combustion, the stoves are expected carbonisation were emissions of pollutants such as CO and PM. |
| Forced draft TEG | Thermoelectric generator. The TEG is a rocket-style stove that converts waste heat into electricity, powering a fan and USB port. It is made of with stainless steel combustion chamber and has a cast-iron bottom and pot support. |

Adapted from Clean Cooking Catalog descriptions <http://catalog.cleancookstoves.org/stoves> (last accessed 25/09/2022) and from ICS descriptions in the studies.
