# Supplementary figures and images for: Improved cookstoves to reduce household air pollution exposure in sub-Saharan Africa: A scoping review of intervention studies
Source: PLoS One. 2023 Apr 27;18(4):e0284908. doi: 10.1371/journal.pone.0284908 (PMC10138283; doi:10.1371/journal.pone.0284908)

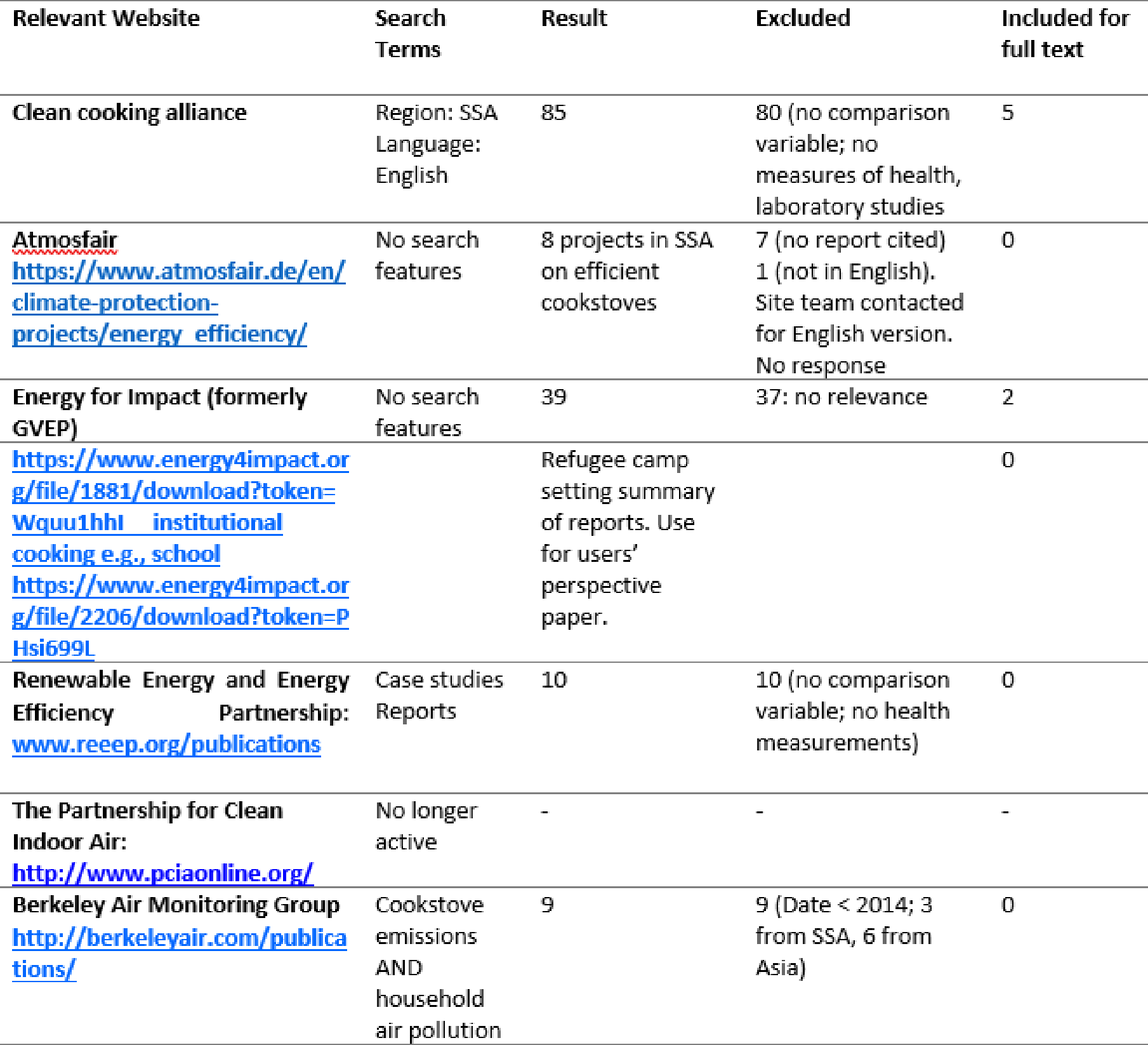

Supplement: S1 Fig — A. Sample of database search terms with results from EMBASE June 2020, July 2021, September 2022. B. Sample of relevant organisation searches and outcomes. (ZIP) [file pone.0284908.s003.zip › S1B_Fig.tif]

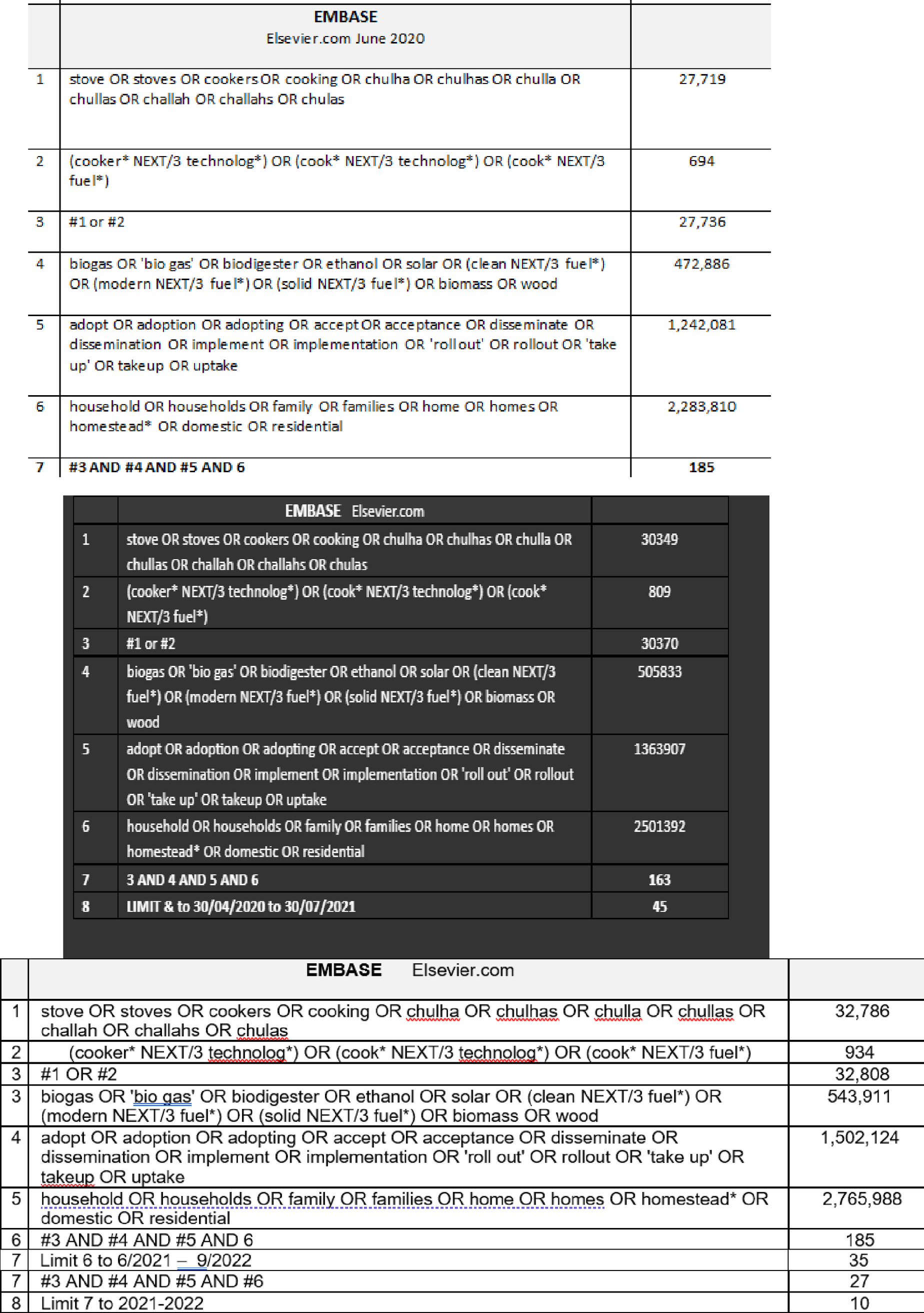

Supplement: S1 Fig — A. Sample of database search terms with results from EMBASE June 2020, July 2021, September 2022. B. Sample of relevant organisation searches and outcomes. (ZIP) [file pone.0284908.s003.zip › S1A_Fig.tif]

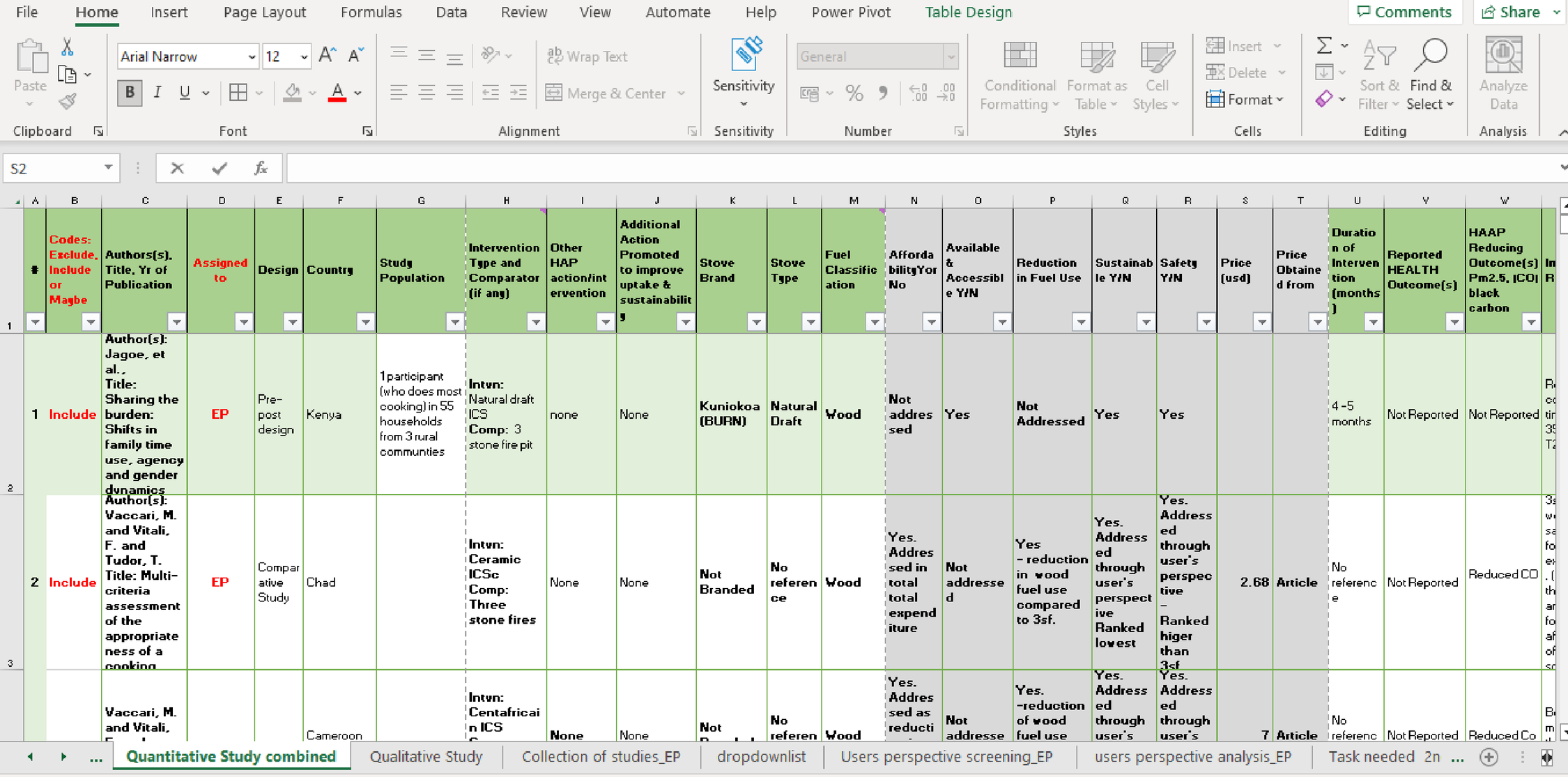

Supplement: S2 Fig — (TIF) [file pone.0284908.s004.tif]

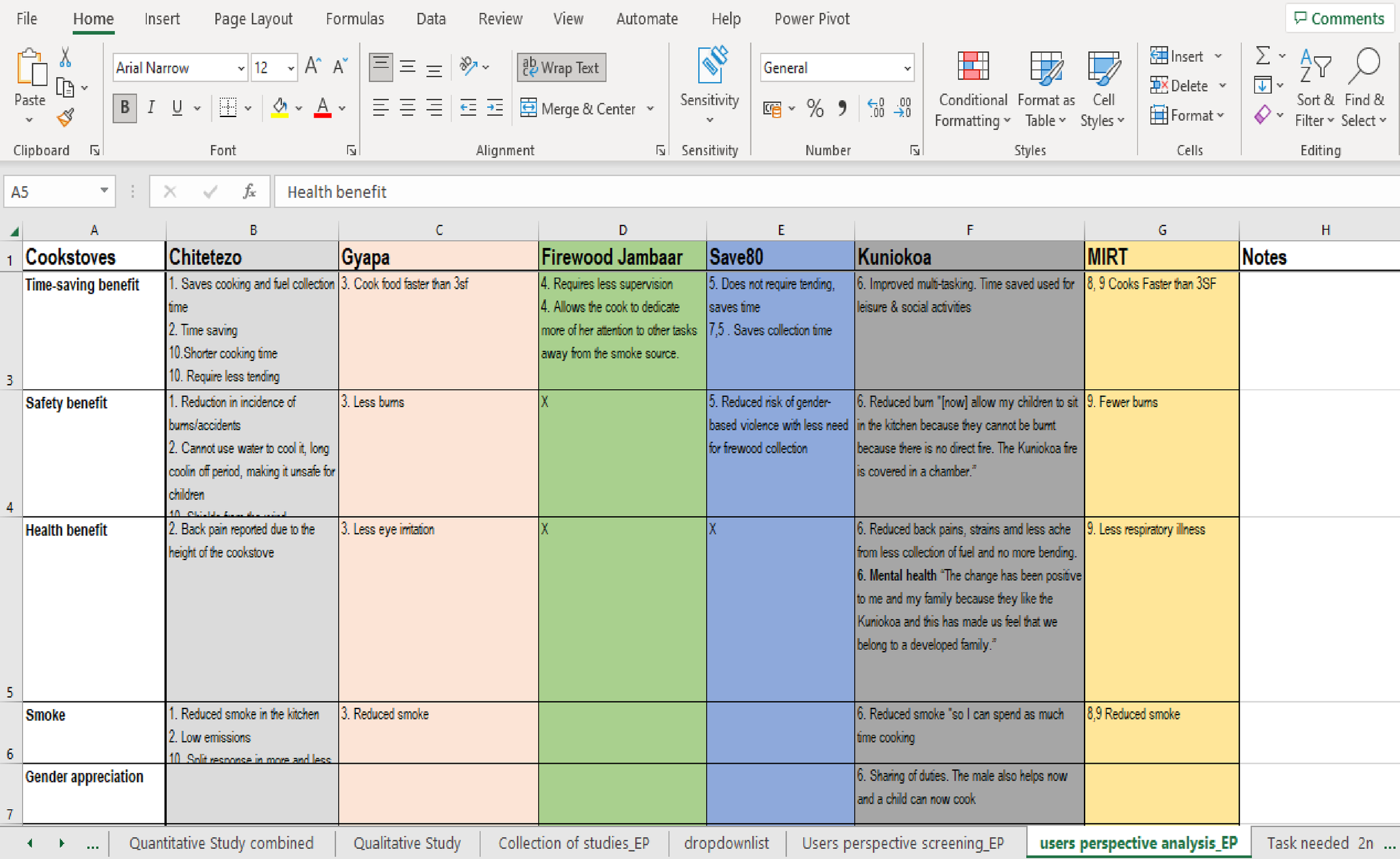

Supplement: S3 Fig — (TIF) [file pone.0284908.s005.tif]

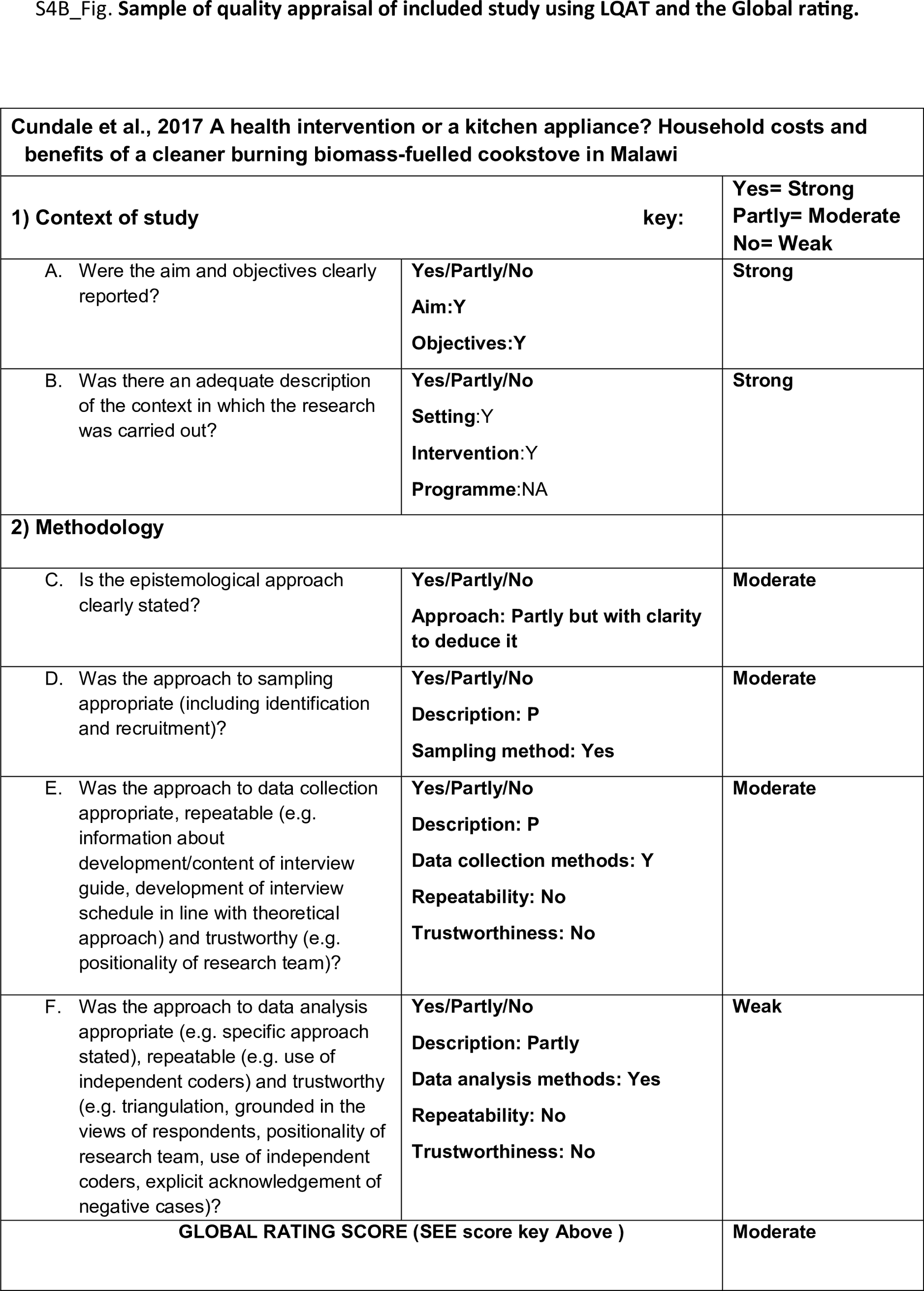

Supplement: S4 Fig — A. Sample of quality appraisal of included quantitative study using LQAT and the global rating tool. B. Sample of quality appraisal of included qualitative study using adapted Hayden et al.’s and the global rating tools. (ZIP) [file pone.0284908.s006.zip › S4B_Fig.tif]

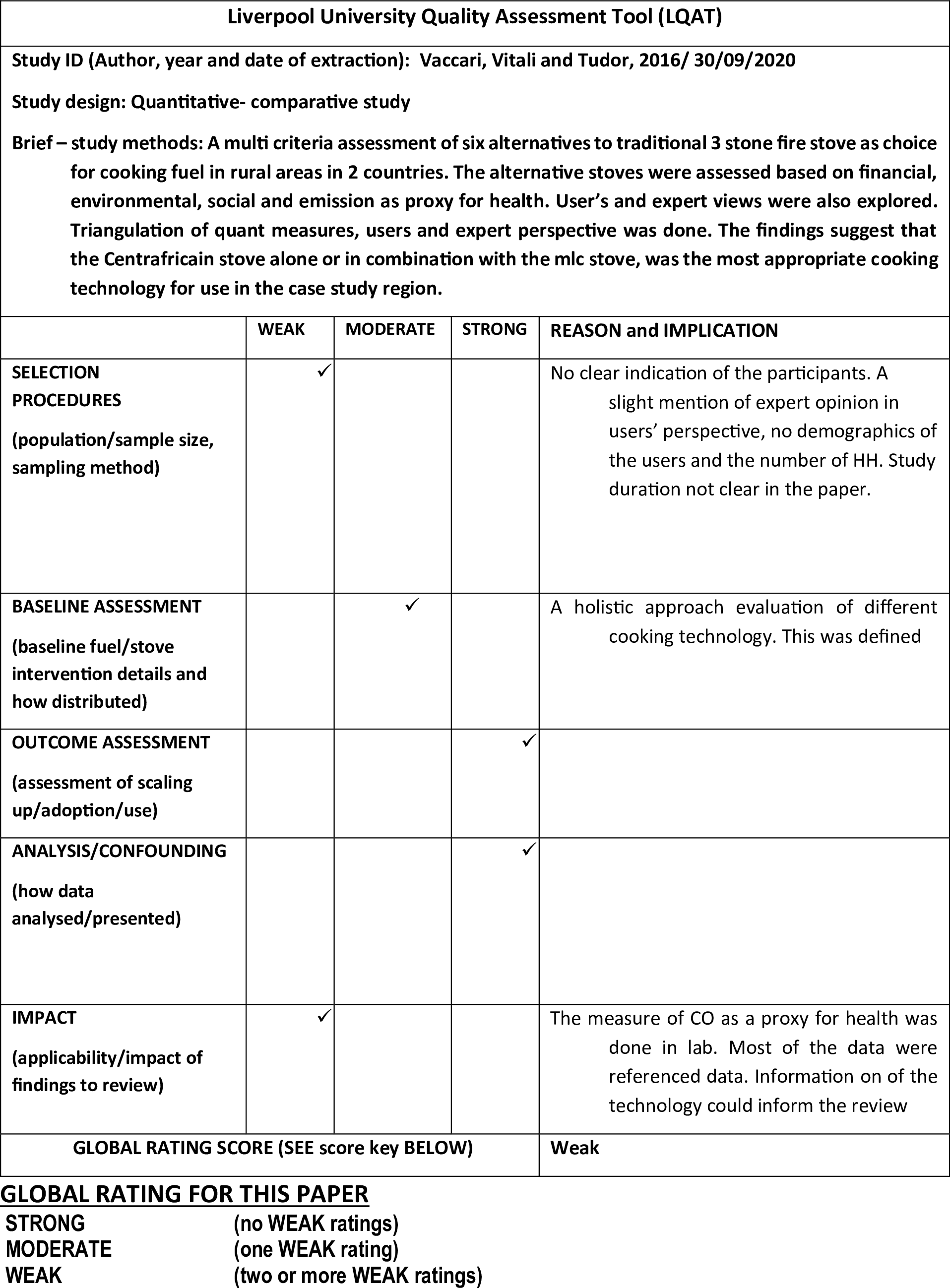

Supplement: S4 Fig — A. Sample of quality appraisal of included quantitative study using LQAT and the global rating tool. B. Sample of quality appraisal of included qualitative study using adapted Hayden et al.’s and the global rating tools. (ZIP) [file pone.0284908.s006.zip › S4A_Fig.tif]
